# Fossilization can mislead analyses of phenotypic disparity

**DOI:** 10.1098/rspb.2023.0522

**Published:** 2023-08-09

**Authors:** Thomas J. Smith, Robert S. Sansom, Davide Pisani, Philip C. J. Donoghue

**Affiliations:** ^1^ Bristol Palaeobiology Group, School of Earth Sciences, University of Bristol, Life Sciences Building, Tyndall Avenue, Bristol BS8 1TQ, UK; ^2^ Department of Earth Sciences, University of Oxford, South Parks Road, Oxford OX1 3AN, UK; ^3^ Department of Earth and Environmental Sciences, University of Manchester, Williamson Building, Oxford Road, Manchester M13 9PL, UK

**Keywords:** disparity, morphology, simulation, fossilization, decay, biostratinomy

## Abstract

Analyses of morphological disparity can incorporate living and fossil taxa to facilitate the exploration of how phenotypic variation changes through time. However, taphonomic processes introduce non-random patterns of data loss in fossil data and their impact on perceptions of disparity is unclear. To address this, we characterize how measures of disparity change when simulated and empirical data are degraded through random and structured data loss. We demonstrate that both types of data loss can distort the disparity of clades, and that the magnitude and direction of these changes varies between the most commonly employed distance metrics and disparity indices. The inclusion of extant taxa and exceptionally preserved fossils mitigates these distortions and clarifies the full extent of the data lost, most of which would otherwise go uncharacterized. This facilitates the use of ancestral state estimation and evolutionary simulations to further control for the effects of data loss. Where the addition of such reference taxa is not possible, we urge caution in the extrapolation of general patterns in disparity from datasets that characterize subsets of phenotype, which may represent no more than the traits that they sample.

## 1. Background

Analyses of disparity seek to elucidate patterns in the evolution of morphological variation and the processes that underlie them. Such analyses have shed light on the emergence of animal body plans (e.g. [1]), mass extinction dynamics [2,3], and the tempo and mode of major diversification events (e.g. [4,5]). An advantage of these methods is their ability to characterize evolution in the absence of a well-resolved phylogeny, which has led to their application to a wide variety of evolutionary questions [6]. Many of these questions centre on how disparity evolves through time (e.g. [7]), thereby necessitating the inclusion of palaeontological data. This is problematic as the processes of decay and preservation inevitably degrade the information content of fossil remains, with different depositional environments inducing different degrees of data loss [8]. Nevertheless, fossil phenotypes are routinely employed as proxies for whole-body (organismal) disparity (e.g. [4,5,9,10–13]). Recent studies have suggested that this practice can be taken one step further, and that for some clades, a subset of the fossilized traits is all that is required to derive meaningful insights into the evolution of organismal disparity within them [14,15]. However, these studies have only considered whether such subsets are representative of all fossilized characters, not entire phenotypes—the majority of which are never preserved even under the most exceptional conditions for fossilization.

Simulation studies have been used extensively to investigate the effect of distributions of missing data on estimates of phylogeny. This includes attempts to invoke fossilization-style filters whereby missing data entries are concentrated in taxa and/or characters [16–19]. Additionally, studies of empirical phylogenetic data have found that fossilization-style filters introduce significant differences in phylogenetic signal, as does partitioning characters by how readily they are fossilized [20–22]. Disparity studies are often based on the same kinds of morphological data but relatively few have directly assessed the impact of missing entries [23–27]. Of those that have, several focus on random data loss [23,26], an unrealistic problem in empirical analyses given the tendency for researchers to be selective in their sampling of morphological variety and the differences in preservation potential between different anatomical elements (e.g. between mineralized and unmineralized tissues [17,28,29]). Others have focused on the effects of non-random data loss [24,25,27]. However, these studies have either induced unrealistically small differences in data loss between groups of organisms [24], used highly taxon-specific re-coding schemes [25], or inappropriately characterized changes in disparity [27]. As such, the impact of biostratinomic processes on morphological disparity remains unclear.

Here, we explore the impact of random and non-random data loss on our ability to accurately quantify morphological disparity. For clarity, we consider data loss to be random when each character score has an equal probability of being lost, and non-random when these probabilities vary. First, we simulate discrete character data along a tree with equal numbers of fossil and extant taxa. Next, we replicate the effects of biostratinomic processes by progressively removing the character scores of fossil taxa. For comparison, we also randomly degrade the simulated data. These data are then analysed with a suite of disparity indices, calculated using the generalized Euclidean distance [30] and the maximum observable rescalable distance [26,31]. The results are then compared to complementary analyses of empirical datasets degraded in the same way to validate their applicability. Taken together, our results demonstrate that both random and non-random data can introduce substantial biases into analyses of morphological disparity. These biases vary considerably between distance metrics and disparity indices. We demonstrate that researchers should include extant taxa where possible, both to mitigate the effects of these biostratinomic processes and shed light on how the data loss they induce (observed and unobserved) has differentially affected fossil taxa and morphological modules. Finally, we caution against the interpretation of trends in disparity obtained from subsets of phenotype as representative of whole organisms.

## 2. Methods

The following section outlines our methodological approach; a more detailed version can be found in the electronic supplementary material of this manuscript. All analyses were conducted using R [32].

### (a) Assembling a generating tree and simulating a base matrix

**Figure 1.**
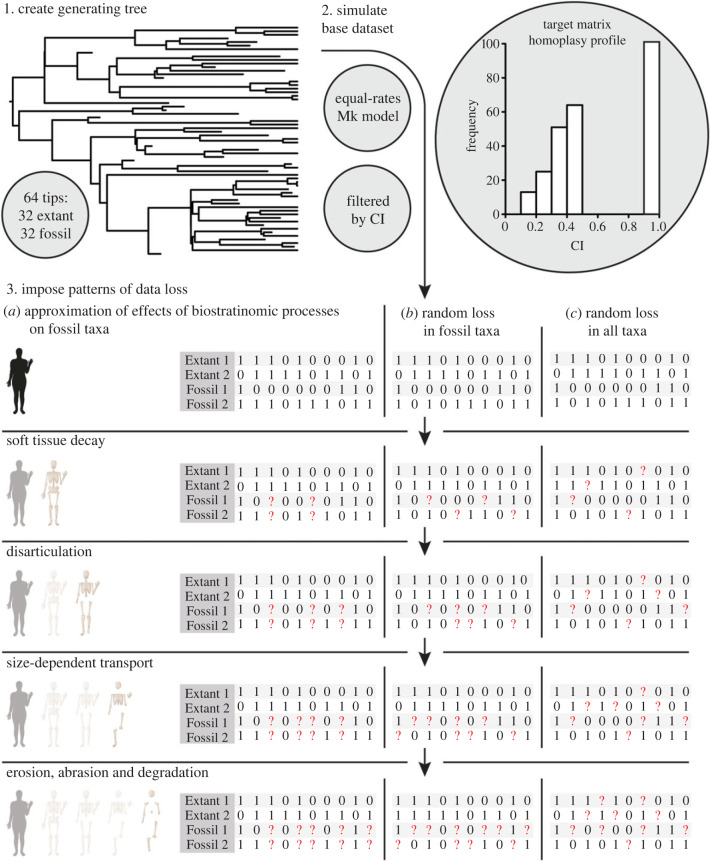
Pipeline used to simulate character data and induce non-random data loss across fossil taxa (*a*), random loss across fossil taxa (*b*) and random loss across all taxa (*c*).

The diversitree tree.bd function [33] was used to generate a 64-tip, fully bifurcating tree with 32 fossil tips, 32 extant tips, and no zero-length branches (figure 1). Data were simulated along the generating tree using a variation of the pipeline employed by Smith *et al*. [34]. This pipeline uses the ape rTraitDisc function to simulate discrete binary character data using a Markovian model [35], specifically an equal-rates model with rate = 0.01. All other rTraitDisc arguments were left in their default settings. In an effort to achieve an empirically realistic distribution of homoplasy [36,37], we simulated the evolution of 254 binary characters (4 : 1 character : taxon ratio) which collectively approximated the per-character homoplasy distribution of the average empirical dataset [38]. Per-character homoplasy was quantified using the consistency index [39], which we calculated using functions from the phangorn R package [40]. Moving forward, we refer to the concatenation of the 254 simulated characters as the ‘original matrix’.

### (b) Introducing missing data

Empirical fossilization processes introduces distinct, non-random distributions of missing data into morphological data as a result of the sequential processes of: (i) soft tissue decay; (ii) disarticulation; (iii) size dependent transport; (iv) erosion, abrasion and degradation [8,29,41]. To approximate the effects of these processes, we induced four increments of data loss in the fossil taxa of the original matrix (figure 1*a*). We began by randomly selecting and converting 43% of characters to missing for all fossil taxa to simulate the decay of soft tissue features, which on average constitute 43% of characters in morphological datasets [21]. We repeated this process 100 times to generate 100 degraded versions of the original matrix. These matrices were then further degraded by converting 60% of the remaining characters to missing for all fossil taxa in 20% increments. This produced 100 four-matrix series across which the proportion of fossil character scores coded as ‘missing’ increased from 43% to 54.4%, 65.8%, and finally 77.2%, which equates to 21.5%, 27.2%, 32.9% and 38.6% of all character scores being lost from each matrix, respectively. These series are referred to as the ‘non-randomly degraded matrices' or simply the ‘first series’ throughout the rest of this study.

We then repeated this process twice over, increasing the randomness with which character scores were selected for removal each time. In the first repetition (hereafter the ‘second series’), data loss was still restricted to the fossil taxa but was less uniform; individual character scores were selected for removal, rather than individual characters (figure 1*b*). In the second (hereafter the ‘third series’), each character score, regardless of whether it belonged to an extant or fossil taxon, had an equal probability of being lost (figure 1*c*). This produced two sets of 100 series across which the proportion of all character scores coded as ‘missing’ increased from 21.5%, to 27.2%, 32.9% and finally 38.6%. We refer to these series as the ‘randomly degraded matrices'. Furthermore, we collectively refer to both the randomly and non-randomly degraded matrices as the ‘degraded matrices’.

### (c) Distance matrix computation and ordination

Pairwise distance matrices were calculated for the original and degraded matrices using the Claddis calculate_morphological_distances function [26]. We used both the original version (ged_type = ‘wills’) of the GED [42] and maximum observable rescaled distance [26,31] so that we could ascertain how the contrasting approaches to accommodating missing data of the two distance metrics affect morphological disparity. Combinations of taxa with non-overlapping character data were identified and the most incomplete taxa removed until a complete matrix could be generated using the Claddis trim_matrix function [26]. Two sets of MORD matrices were generated: one for pre-ordination disparity characterization and another for post-ordination. The latter were first transformed using a square root term and then, along with all others intended for ordination, further transformed via the application of the Cailliez correction [43]. These transformations help ensure the resulting ordinations are Euclidean [30]. Distance matrices were ordinated via principal coordinates analysis, otherwise known as classical multidimensional scaling, which we conducted using the ape pcoa function [35].

### (d) Characterizing disparity

We used both pre-ordination and post-ordination indices to characterize the disparity of the original and degraded matrices to enable an assessment of the impact of ordination. For pre-ordination characterization, we used the mean pairwise distance, maximum pairwise distance and mean pairwise extant–fossil distance, the latter of which was defined as the average of the morphological distances separating the extant taxa from the fossils. To characterize disparity post-ordination, we used the sum of variances, sum of ranges and the mean Euclidean distance between the extant and fossil centroids. Index values were verified using functions from the dispRity R package [44].

Two sets of analyses were employed using these indices. The first used all six to characterize the effects of fossilization on the disparity of matrices containing both extant and fossil taxa (hereafter described as ‘mixed’ matrices’). One hundred 64-taxon subsamples were randomly drawn with replacement from the distance matrices and ordinations of each series, their disparity subsequently characterized using pre- and post-ordination indices, respectively. The median disparity values for each set of 100 subsamples were then isolated. This yielded 100 values of each disparity index (one per distance matrix/ordination) for each combination of missing data percentage and missing data type (random versus non-random). These values were then compared to the median disparity values of the original matrix, which were derived in the same way. To make the changes in disparity associated with data loss easier to interpret, we present them as the ratio between the median disparity of the original and degraded matrices.

The second set of analyses applied the same approach as the first with three key differences. Firstly, as the aim of these analyses was to quantify how data loss affects the disparity of matrices wholly composed of fossil taxa (hereafter described as ‘fossil matrices’), all 32 extant taxa were removed from each distance matrix and ordination prior to subsampling. Accordingly, the subsamples drawn were composed of 32 taxa, not 64. Finally, as the absence of extant taxa made it impossible to calculate the mean pairwise extant–fossil distance and mean distance between the extant and fossil centroids, the effects of data loss on fossil disparity were characterized using the mean pairwise distance, maximum pairwise distance, sum of variances and sum of ranges.

### (e) Empirical validation

To establish the biological realism of the findings from our simulations, we repeated our analyses on subsets of the 4541-character mammal dataset first assembled by O'Leary *et al*. [45]. Following the approach of O'Reilly & Donoghue [41], we derived 2545- and 254-character subsets of the dataset, which we refer to as the ‘large mammal matrix’ and ‘small mammal matrix’ going forward. This allowed us to explore how dataset size interacts with the effects of data loss. Both subsets contained the same 66 taxa, of which only 20 were fossils. These subsets were remarkably complete for empirical datasets, as less than 5% of all character scores and less than 12% of all fossil character scores were coded as missing. Nevertheless, their incompleteness meant that all measures of disparity derived from them incorporated small amounts of missing data. It also meant that the proportion of the remaining fossil character scores coded as ‘missing’, not the proportion of all, increased from 43% to 54.4%, 65.8% and 77.2% in each series of degraded matrices we derived from these subsets. Given the extant : fossil taxon ratio (46 : 20), this equated to 13%, 16.5%, 20% and 23.5% of the remaining character scores being changed to ‘missing’, respectively.

## 3. Results

All ratios reflect the difference between the disparity of the original, small mammal and large mammal matrices, and that of the degraded matrices derived by removing data from them. Ratios above 1 represent overestimations of disparity, whereas those below 1 are underestimations. While our results focus on the average change in disparity induced through incrementally removing data from 100 copies of the same matrix, it is worth noting that different patterns of change were recovered between individual series. Some exhibited unidirectional, constant changes in disparity, while the changes induced in others varied in their polarity and magnitude between increments of data loss.

### (a) Analysing extant and fossil taxa together

**Figure 2.**
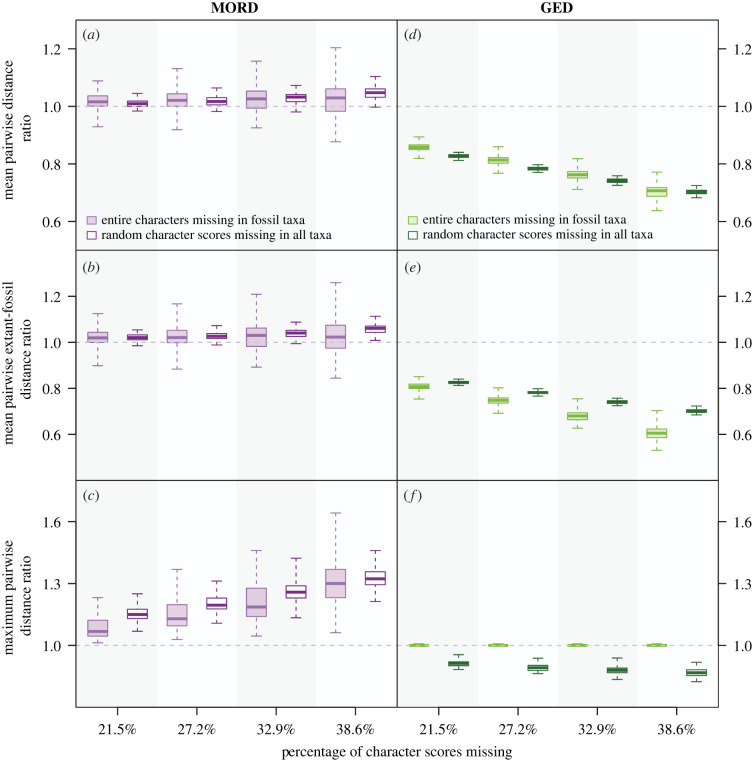
How different patterns of data loss affect the mean pairwise distance (*a*,*d*), mean pairwise extant–fossil distance (*b*,*e*), and maximum pairwise distance (*c*,*f*) of the first and third series of simulated mixed matrices. Each boxplot summarizes 100 original-degraded matrix ratios: the whiskers encompass the range, the box delimits the interquartile range, and the central line denotes the median. Ratio 1 : 1 (no change) = grey dashed line. GED = generalized Euclidean distance. MORD = maximum observable rescaled distance.

**Figure 3.**
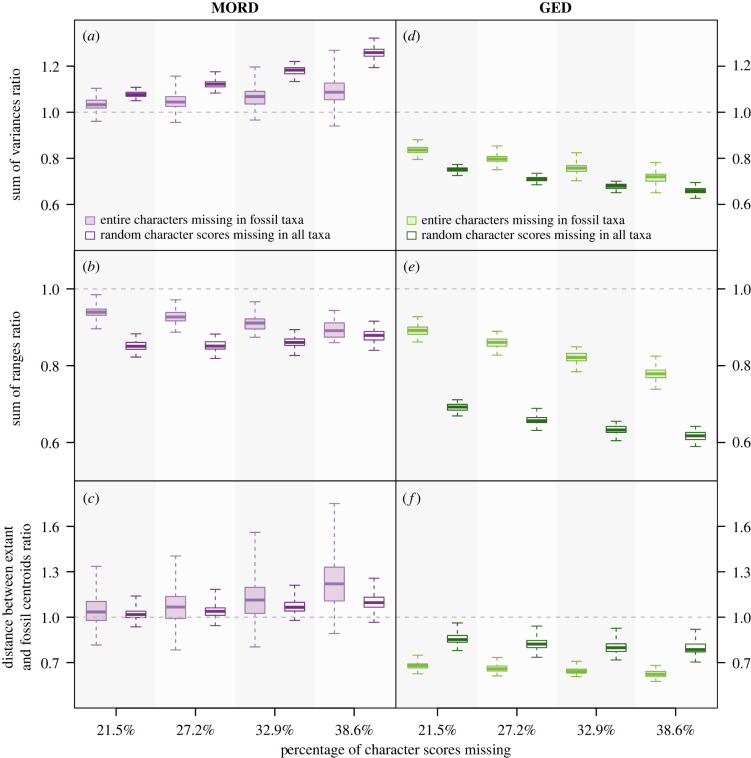
How different patterns of data loss affect the sum of variances (*a*,*d*), sum of ranges (*b*,*e*), and distance between the extant and fossil centroids (*c*,*f*) of the first and third series of simulated mixed matrices. Each boxplot summarizes 100 original-degraded matrix ratios: the whiskers encompass the range, the box delimits the interquartile range and the central line denotes the median. Ratio 1 : 1 (no change) = grey dashed line. GED = generalized Euclidean distance. MORD = maximum observable rescaled distance.

Overall, the average change in disparity induced by randomly removing character scores from mixed matrices exceeded that produced by non-random data loss when MORD was employed, except for the distance between the extant and fossil centroids (figures 2 and 3). When 38.6% of character scores were removed, the median changes rooted in random data loss ranged from decreases of over 12% to increases of over 32%, while those resulting from non-random data loss ranged from decreases of just over 10% to increases of just over 30% (electronic supplementary material, table S1). However, the maximum changes in all three pre-ordination indices were more extreme in the non-randomly degraded matrices than the randomly degraded matrices, with the maximum pairwise distance increasing by more than 70% (electronic supplementary material, table S1). This was also true for the changes induced through data loss in the distance between extant and fossil centroids.

On average, random data loss in mixed matrices induced greater decreases in GED-based mean pairwise distance, maximum pairwise distance, sum of variances and sum of ranges than non-random (figures 2 and 3). The inverse was true for the mean extant–fossil pairwise distance (figure 2*e*) and the distance between the extant and fossil centroids (figure 3*f*), although the maximum decrease recovered in the former through the removal of fossil characters (−46.9%) exceeded that produced by random data loss (−31.5%; electronic supplementary material, table S1). When 38.6% of character scores were converted to missing, the median effects of random data loss on disparity ranged from −13.3% to −38.3%, while the non-random removal of fossil characters either had no effect (maximum pairwise distance) or induced decreases of 22.1–39.5% (electronic supplementary material, table S1).

Except for the sum of ranges, MORD-based disparity generally increases as data are lost from a mixed matrix (figures 2 and 3). By contrast, GED-based disparity generally decreases. On average, the fidelity of GED deteriorated to a greater extent with data loss than MORD. When GED was used, the most incomplete degraded matrices presented median disparity values up to 39.5% smaller than that of the original matrix, whereas the most extreme change recovered when MORD was employed was an increase of 32.3% (electronic supplementary material, table S1). However, the effects of data loss on disparity were much more varied when MORD was employed over GED, as across all indices apart from the sum of ranges, the range and variance of the changes quantified using the former surpassed those employing the latter (electronic supplementary material, table S1).

Across almost every combination of distance metric and disparity index, the direction of change did not differ between random and non-random data loss, with two exceptions (figures 2 and 3). The first occurred when changes in maximum pairwise distance were calculated using GED (figure 2*f*); non-random data loss did not produce any changes in disparity, whereas the random removal of character scores produced a decline. The other exception occurred when the sum of ranges was calculated using MORD (figure 3*b*). While both types of data loss produced an overall decrease in this measure of disparity, the extent to which the randomly degraded matrices differed from the original decreased as more data were removed, whereas it increased in the non-randomly degraded matrices. The polarity of the average change in disparity did not differ between the two types of data loss. However, a much greater variety of changes was induced when entire fossil characters were removed, MORD was employed, and all indices (excepting sum of ranges) were used to quantify disparity (figures 2 and 3). The broadest distribution of changes was recovered from the most incomplete non-randomly degraded matrices (electronic supplementary material, table S1). A much weaker version of this relationship was recovered when GED was used to calculate both types of mean pairwise distance (figure 2*d,e*), the sum of variances (figure 3*d*) and sum of ranges (figure 3*e*). The maximum pairwise distance (figure 2*f*) and the distance between the extant and fossil centroids (figure 3*f*) presented the opposite relationship.

Pivoting between pre-ordination and post-ordination indices did not consistently alter the effects of missing data on disparity (figures 2 and 3; electronic supplementary material, table S1).

### (b) Analysing fossil taxa in isolation

**Figure 4.**
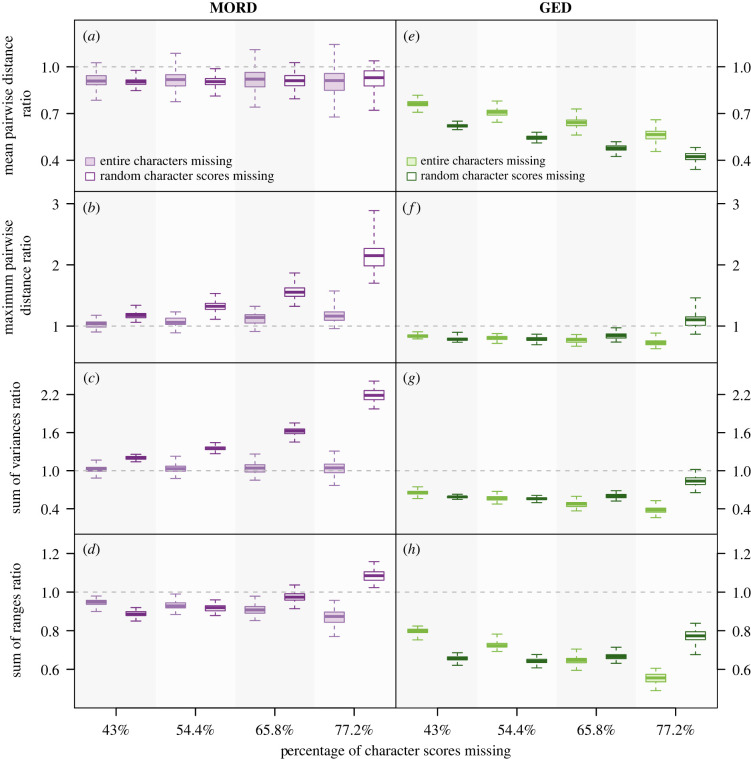
How different patterns of data loss affect the mean pairwise distance (*a*,*e*), maximum pairwise distance (*b*,*f*), sum of variances (*c*,*g*) and sum of ranges (*d*,*h*) of the fossil components (the fossil matrices) of the first and second series of simulated matrices. Each boxplot summarizes 100 original-degraded matrix ratios: the whiskers encompass the range, the box delimits the interquartile range, and the central line denotes the median. Grey dashed line = ratio 1 : 1 (no change). GED, generalized Euclidean distance; MORD, maximum observable rescaled distance.

Random and non-random data loss induced comparable patterns of change in the MORD-based mean pairwise distance, maximum pairwise distance, and sum of variances and GED-based mean pairwise distance of fossil matrices (figure 4). However, they caused different patterns of change in other indices. While both types of data loss generally produced degraded matrices with smaller GED-based maximum pairwise distances (figure 4*f*) and sums of variances (figure 4*g*) than the original matrix, the extent to which the randomly degraded matrices differed decreased as more character scores were removed, whereas it increased in the non-randomly degraded matrices. The sum of ranges of the degraded matrices also presented this dichotomy, regardless of the distance metric employed (figure 4*d,h*). In two indices, the final increment of random data loss we induced caused the direction of change to flip; both the MORD-based sum of ranges (figure 4*d*) and GED-based maximum pairwise distances (figure 4*f*) of the most incomplete randomly degraded matrices exceeded that of the original matrix.

Non-random data loss produced a greater variety of changes in mean pairwise distance, whereas the range of and variance in maximum pairwise distances recovered was higher in the randomly degraded fossil matrices (electronic supplementary material, table S2). The changes in sum of ranges and sum of variances did not vary as consistently between the two types of data loss. When MORD was employed, non-random data loss produced a greater variety of changes, while GED-based sums of ranges and sums of variances varied more substantially under random data loss (figure 4, electronic supplementary material, table S2). In most cases, the relative magnitudes of the changes in disparity caused by different types of data loss varied between each combination of disparity index and distance metric. Only the sum of ranges presented any consistency, as non-random data loss induced a greater change than random across both distance metrics (figure 4; electronic supplementary material, table S2). This was also the case for the MORD-based mean pairwise distances, and GED-based maximum pairwise distances and sums of variances, whereas the opposite was true when these indices were calculated using the other distance metric (GED and MORD respectively). In the most incomplete fossil matrices (77.2% of character scores missing), the median changes in disparity caused by random data loss ranged from −47.7% to +218.7%, whereas in those subjected to non-random data loss, they ranged from −61.9% to +16.3% (electronic supplementary material, table S2).

When fossil data are degraded and analysed in isolation, the differences between MORD and GED become less defined (figure 4). GED-based measures of disparity were generally lower for the degraded matrices than the original matrix. Similarly, the MORD-based mean pairwise distances (figure 4*a*) and sums of ranges (figure 4*d*) of the degraded matrices were lower than that of the original matrix. By contrast, MORD-based maximum pairwise distances (figure 4*b*) and sums of variances (figure 4*c*) increased with data loss. In the absence of complete taxa, the fidelity of MORD deteriorated to a greater extent than GED, with the sum of variances of the most incomplete matrices doubling when calculated using the former (electronic supplementary material, table S2). Furthermore, using MORD also presented a greater range and variance in the changes in mean pairwise distance, maximum pairwise distance and sum of variances (figure 4; electronic supplementary material, table S2). This was also partially true for the sum of ranges of the degraded matrices, although the most incomplete matrices varied more when GED was used (figure 4*h*). The relative magnitude of these changes varied between indices. GED-based mean pairwise distances and sums of ranges diverged more from the original matrix than their MORD-based counterparts, while the inverse was true for the maximum pairwise distance and sum of variances (electronic supplementary material, table S2). The effects of data loss on disparity peaked in the most incomplete matrices, with the median changes ranging between −12.6% and +118.7% for MORD, and −61.9% and +10.3% for GED (figure 4; electronic supplementary material, table S2).

As with the analyses including extant taxa, the effects of data loss on the disparity of fossil matrices did not consistently vary between the pre-ordination and post-ordination indices. Rather, the effects not attributable to the type of data loss induced or distance metric used were generally index-specific (figure 4; electronic supplementary material, table S2).

### (c) Analyses of empirical data

Equivalent analyses of the mammal matrices presented comparable results to those recovered from analyses of the simulated matrices, with one notable exception. When the mammal matrices were randomly degraded and their sum of ranges calculated using MORD (see electronic supplementary material for figures), disparity gradually decreased, whereas in the simulated data analyses, this combination of data loss, distance metric and index produced a net decrease in disparity but one that lessened as the amount of data removed increased (figures 3*b* and 4*d*). Additionally, analyses of both mammal matrices resulted in smaller changes in disparity across all six indices as character scores were removed, but as the increments of data loss were smaller than in the analyses of the original matrix, this was expected. When GED was employed, no consistent differences were recovered between the small and large mammal matrices, despite the differences in taxon–character ratio. However, MORD characterized smaller changes in disparity in the large degraded mammal matrix than the small degraded mammal matrix (see electronic supplementary material for figures).

## 4. Discussion

### (a) Implications for distance metric and disparity index selection

GED and MORD accommodate missing data in fundamentally different ways; GED replaces missing dissimilarities with the mean of those that can be calculated, whereas MORD scales the distance values derived from the available data by the number of characters that could be compared for each taxon pair. This difference is reflected in how they change as data are lost. Our results confirm that using GED in analyses of incomplete datasets leads to substantial misrepresentations of disparity [23,24,26]. Furthermore, they corroborate the negative relationship recovered between GED-based disparity and data loss in previous empirical analyses [23,24]. This relationship is to be expected as all character dissimilarities are squared as part of the calculation of GED. This transformation does not affect observed dissimilarities, as all are either 0 or 1 in binary character datasets, but it almost always diminishes the mean dissimilarities that are used in place of incalculable dissimilarities, as most fall between 0 and 1. Where these values replace a dissimilarity that would have been 1, this squaring exacerbates the reduction in dissimilarity. Conversely, where they replace a dissimilarity that would have been 0, the increase in dissimilarity between two taxa is minimized by the squaring. This causes GED to tend towards zero as character scores are removed. This mirrors the effect of data loss on distance metrics which simply ignore incomparable character pairs, such as the raw Euclidean distance (RED) and its derivatives [27]. As GED was designed as an extension to the RED that accommodates missing data instead of just ignoring it, the fact that these distance measures are similarly affected by data loss calls into question the value of using GED over RED.

We find further support for the view that MORD generally outperforms GED in accurately characterizing the disparity of a clade after biostratinomic processes have degraded entire characters [23,24,26] but not when data loss is effectively random. The invariance in mean pairwise distance and increase in sum of variances we recover as character scores are non-randomly removed matches the results of previous analyses [24]. However, the decrease in sum of ranges we recover does not [24]. The cause of these differences is unclear, but they likely lie in the heterogeneous character compositions of the underlying datasets. This interpretation is supported by the fact that we recover the same positive relationship between sum of ranges and data loss in our empirical analyses as Lehmann *et al*. [24]. Similarly, it is not immediately apparent why different indices, particularly those derived post-ordination, are affected by data loss in different ways when calculated using MORD. Further theoretical work is needed to resolve this issue. What is apparent, however, is that the increase in variance in MORD-based estimates of disparity is rooted in the increase in the heterogeneity of the characters being compared for each pair of taxa in each degraded matrix. As each degraded matrix differed in its distribution of missing data, so too did the comparable characters for each taxon pair within it. Different sets of comparable characters produce different values of MORD and thus different estimates of disparity. As such, it is to be expected that MORD-based estimates of disparity will become less precise with increasing data loss.

There were other conflicts between our results and those of previous studies. As the sum of variances and mean distance from centroid are deterministically related, their covariance is to be expected [34]. As such, the invariance under data loss of the MORD-based mean distances from centroid reported by Sutherland *et al*. [23] conflict with the changes in MORD-based sum of variances we recovered. This could be a consequence of ignoring negative eigenvalues rather than correcting for them [23]. However, this explanation remains untested. Another conflict lies in the decrease in crinoid pairwise distances as data were removed recovered by Deline & Thomka [25] using the Gower coefficient (GC), the distance metric that MORD is based upon. In analyses of binary character data, there is no difference between how these two metrics are calculated and so they should behave the same way under data loss. As such, the results presented by Deline & Thomka [25] conflict with the relative invariance in MORD-based mean pairwise distance we recovered, although this almost certainly reflects differences in the composition of the underlying datasets.

At first glance, our results could be taken as further evidence that pre-ordination indices of disparity outperform post-ordination approaches [23,24,26,46]. However, we found that this only applies to mean pairwise distances. We followed Gerber [46] in using the maximum pairwise distance as a pre-ordination analogue of the sum of ranges and found it to be highly susceptible to the effects of missing data. Additionally, we found that it responded differently to data loss than its supposed post-ordination counterpart. These results lay bare the deficiencies of pre-ordination disparity measures; while there are numerous post-ordination indices for characterizing different aspects of morphological disparity [47], there are relatively few pre-ordination alternatives. Future studies would do well to focus on addressing this deficiency.

### (b) Implications for other measures of disparity

Analyses of the effect of taphonomic compaction on trilobites using geometric morphometrics have shown that the resulting distortion of landmarks can effectively treble the spread of points in morphospace [48]. This result, coupled with the general correlation between estimates of disparity derived from categorical and continuous data [49–52], suggest that our results have bearing on quantitative analyses of disparity more generally, although the exact effects of continuous data loss have yet to be characterized. When they are, it is likely that they will behave in a similar way to measures of categorical disparity derived using GED, as the ‘thin-plate spline’ and ‘regression’ methods typically used to account for missing landmarks do so by estimating their location based upon the remaining available data [53], which is similar to how the distance metric accommodates missing data. Our results also have implications for qualitative assessments of morphospace occupation. Typically, these focus on the relative positions of different clusters of taxa in two or three dimensions derived through ordination. Our analyses demonstrate that data loss can drastically change these distributions by causing the distances separating fossil and extant clades to inflate (when MORD is employed) or contract (when GED is employed) by over 40%, depending on the distance metric used. These issues will manifest whenever missing data is unevenly distributed amongst clusters and/or clades, compounding the well-documented issues with using visualizations of morphospace occupation for interpreting relative disparity [46].

### (c) Past problems and future solutions

The regime of non-random data loss we used is just one of the many plausible outcomes of fossilization. Of the others that have been applied in studies of disparity, the taxon-specific re-coding scheme of Deline & Thomka [25] probably introduced the most realistic pattern of data loss, as the probabilities of loss assigned to each character reflected the echinoderm body plan the sampled features belonged to and its propensity for disarticulation and degradation. However, such a scheme cannot be extended to other taxa without a comparable taxon-specific knowledge of their taphonomy; a worthy goal when studying the evolutionary histories of specific clades but an impractical one when seeking to quantify the effects of decay and preservation on disparity more generally.

The linkage algorithm introduced by Smith *et al*. [27] appears to resolve this issue by using the fossil components of mixed matrices to derive probabilities of loss that can be applied in the artificial degradation of the extant components. However, their analyses of the resulting patterns in disparity were compromised by their decision to use the Manhattan distance, an unbounded metric which simply ignores dissimilarities rendered incalculable due to missing character scores. Predictably, these distances collapsed as character scores were removed [27]. As MORD and GED handle missing data differently, these results do little to inform expectations of the impact of data loss in contemporary analyses of disparity. A simple solution to this issue would be to analyse data degraded by the linkage algorithm using either of these metrics, although such analyses would still be limited to mixed datasets with well-sampled extant and fossil components. This limitation could be overcome by the development of a framework capable of simulating contingent character evolution, which would allow the algorithm to be applied in analyses of exclusively palaeontological data.

Lehmann *et al*. [24] came closest to quantifying the general effects of non-random data loss on morphological disparity. However, their study is compromised by the unrealistically small differences (40%) in missing data that they introduced. On average, 43% of morphological data relates to soft tissue features, which rapidly decay after death [20]. Factor in data loss associated with other biostratinomic processes, and it is perhaps unsurprising that over 70% of fossil character scores are missing in some mammal datasets [54]. These data loss can be even more severe in extinct clades: the average completeness of the skeletal and soft tissue fossil records for acanthodians sits at 14% and 18%, respectively [55]. Our study is similar in approach to that of Lehmann *et al*. [24] but crucially, we introduced much more realistic differences in data loss between our groups of taxa, even if the most extreme differences (77.2% of fossil data versus 0% of extant) were, perhaps, still too conservative. Except for testing even more extreme differences, this approach could be improved by a more granular understanding of the composition of empirical morphological data matrices in terms of the average apportioning of characters between different anatomical systems. If this knowledge could be combined with estimates of the average amount of character data lost from the remains of different anatomical systems during each stage of the fossilization process (e.g. [28]), then future studies could approximate the effects of each stage on morphological data with much greater accuracy using a framework similar to the one used herein.

### (d) Caution is needed when using fossils as proxies for organismal disparity

Many analyses of disparity are solely based on fossil data by necessity (e.g. [4,5,9,10–13]). Others have opted to remove or ignore extant data to foster equivalence in the sampling of clades through time (e.g. [7,27]). These studies implicitly assume fossil anatomical variety to be an adequate proxy for organismal disparity, even though some fossil samples are demonstrably poor proxies for the disparity of others [23]. Sutherland *et al*. [23] demonstrated this by analysing ichthyosaur disparity through time with and without exceptionally well-preserved specimens recovered from lagerstätten, from which they recovered distinct patterns in taxon distance from centroid through time. Hughes *et al*. [7] took this approach one logical step further, excluding data from extant taxa because of concerns that the evolutionary histories of extant clades are, by definition, incomplete.

Our analyses demonstrate that this approach is compromised by the incomplete sampling of phenotype that results from analyses of fossil taxa alone. MORD-derived indices of disparity were the most stable measures we tested, with most changing by around 10% or less on average through the most intense regimes of non-random data loss we simulated. However, the range of changes recovered increases beyond this threshold as the amount of data loss increases, peaking when fossils are analysed in isolation. Specifically, MORD-based indices have the potential to underestimate the disparity of a group by more than 30% or overestimate it by more than 60% due to the loss of characters in fossil taxa. Under random data loss, the fidelity of MORD worsens, especially when used to calculate the maximum pairwise distance or sum of variances of a sample. These indices increased by 115% and 118%, respectively, in the most incomplete randomly degraded matrices. Most palaeontological datasets do not include soft tissue characters, which constitute an average of 43% of the morphological datasets that do sample them. Therefore, the higher levels of missing data introduced in this study (e.g. 77.2% of fossil character scores) are likely the most realistic when the effects of physical biostratinomic processes and non-uniform preservation between sites are also accounted for [10,23,56]. Factoring in the unavoidable variation in rates of preservation between environments [8] and through time [57], it is clear that disparity-through-time analyses that do not explicitly account for missing data have the potential to misrepresent evolutionary patterns in total organismal disparity. Given the prevalence of the distance metrics and indices, we tested in contemporary analyses of disparity (e.g. [1,2,4,5,7,9–13]), it is likely that this potential has been realized on numerous occasions, casting doubt on our understanding of landmark evolutionary events such as the fish-tetrapod transition [4] and the rise and fall of the dinosaurs [5,58], as well as the validity of the widely accepted view that clades tend to maximize their disparity early in their evolutionary histories [1,7]. As most datasets that combine palaeontological and neontological data do not present early high disparity [7], the pervasiveness of this pattern in extinct clades may have been exaggerated by uneven data loss in fossil taxa.

### (e) Analyses of parts do not capture the disparity of whole organisms

The extrapolation of general evolutionary insights from subsets of morphological data in analyses of disparity is not limited to using fossil anatomies as proxies for entire organisms. Recent studies have suggested that parts of organisms can be used to adequately represent the disparity of the whole [15,59], despite most morphological traits differing in evolutionary rate and mode (e.g. [60–62]). Specifically, Hopkins [14] found cranidial shape be an adequate proxy for their sampling of species-level trilobite disparity, while the analyses of Deline & Ausich [15] indicated that only 20% or so of the characters they sampled were required to capture most of the properties of the crinoid morphospace they generated. Our results caution against this kind of extrapolation, as it only serves to compound the effect of data loss on perceptions of disparity. It also fails to account for the non-random differences in phylogenetic signal between different character types [20,21,63–66]. As different parts of organisms imply different evolutionary histories, it is illogical to assume the opposite in analyses of disparity. If the morphological variety of fossil species generally misrepresents total organismal disparity, then it follows that subsets of fossil anatomy will do the same for whole-fossil disparity.

While we are far from being able to definitively describe the effects of decay and preservation on patterns of disparity, our results suggest that extrapolating the organismal disparity of a clade from palaeontological data is fraught with risk and uncertainty. This is partially because such datasets are often rife with missing entries, the effects of which are readily constrained through simulation, but also because sampling subsets of fossil anatomy often results in ‘hidden’ losses, features lost to biostratinomic processes that go uncodified. Understanding how these hidden losses are distributed across subsets of taxa and morphological features is essential if one is to be used as a proxy for all. However, these distributions are unknowable without a point of reference. If the intent is to study the evolution of organismal disparity within a group, the reference point must be a complete organism, unaltered by the effects of decay and other biostratinomic processes. Such additions cannot ‘complete’ morphological datasets, for it is practically impossible to codify every aspect of a set of phenotypes even if the organisms they belong to are all extant, but they can reveal how hidden losses are distributed amongst fossil taxa. In doing so, they facilitate simulations that can constrain the plausible range of patterns in organismal disparity implied by the data. Practically, such additions can only be made in analyses of clades with extant descendants. Studies of whole-fossil disparity impose more achievable requirements; the point of reference need only preserve the missing elements one intends to constrain the evolution of. However, our results suggest that this may be the limit of the explanatory power of such analyses; subsets of fossil anatomy will not reliably recover the same patterns in the evolution of disparity as datasets composed of characters more representative of the whole organism. How the relative disparity of crinoid subclasses changes depending on the body regions emphasized during sampling reflects this [15]. As such, it is best to view any correlations between the disparity of fossilized parts and whole organisms [14,15] as fortuitous, rather than manifestations of a general evolutionary phenomenon.

It is important to recognize that, in many cases, it is not possible to elucidate how hidden losses are distributed amongst fossil taxa. Where extant representatives are lacking and the fossils that remain are inconsistently preserved, the simplest solution is to resist the temptation to derive general trends from analyses of subsets of anatomy, whether they be fossils or parts of fossils, and accept that the results reflect no more than the aspects of organismal biology they capture. Ancestral state estimation approaches can facilitate this type of analysis by filling in the gaps created by the non-preservation of taxa [67] and character scores [68] in particularly incomplete datasets. If the extrapolation of more general trends from anatomical subsets is a necessity, the results of this study can be used to provide coarse error bars for the estimates of disparity derived. However, it is better if such palaeontological datasets are restricted to analyses of the specific features and modules they sample, where the effects of data loss can be accounted for, and confidence placed in the trends that are borne out.

## 5. Conclusion

Both random and structured data loss can change perceptions of morphological disparity. How they change depends on the distance metric and disparity index employed, as well as how missing data are distributed across a dataset. However, in most cases pre-ordination indices calculated using MORD change the least as data are lost. In analyses of palaeontological datasets, the addition of extant taxa mitigates the effects of fossilization in two ways; it minimizes the deviations in disparity caused by decay and preservation, and reveals the patterns of data loss induced by these processes that would otherwise go uncodified. By extension, well-preserved fossils lessen the impact of missing data on analyses of subsets of fossilized anatomies and highlight how biostratinomic processes differentially degrade different morphological modules. Understanding these hidden patterns of data loss is an essential first step towards drawing general evolutionary trends from subsets of morphology, as it facilitates the use of simulations that can constrain their impact and thus control for the effects of taphonomy and other deleterious processes. In many cases, however, this is not possible due to a lack of extant descendants and/or well-preserved fossils. While our results can provide error bars that coarsely constrain the plausible range of patterns in organismal disparity implied by subsets of anatomy, our findings caution against this kind of extrapolation. Where the distributions of missing data introduced by structured data loss are unknown, as is often the case in palaeontological datasets, interpretations of patterns in disparity are best limited to the evolution of the sampled features.

## Data Availability

Data available from the Dryad Digital Repository: doi:10.5061/dryad.x69p8czmn [69]. Additional information is provided in electronic supplementary material [70].
